# Harmonizing Dietary Exposure of Adult and Older Individuals: A Methodological Work of the Collaborative PROMED-COG Pooled Cohorts Study

**DOI:** 10.3390/nu16223917

**Published:** 2024-11-16

**Authors:** Federica Prinelli, Caterina Trevisan, Silvia Conti, Stefania Maggi, Giuseppe Sergi, Lorraine Brennan, Lisette C. P. G. M. de Groot, Dorothee Volkert, Claire T. McEvoy, Marianna Noale

**Affiliations:** 1 Institute of Biomedical Technologies, National Research Council (CNR), Via Fratelli Cervi 93, 20054 Segrate, Milano, Italy; federica.prinelli@itb.cnr.it (F.P.); silvia.conti@itb.cnr.it (S.C.); 2 C. Mondino National Institute of Neurology Foundation, IRCCS, Via Mondino, 2, 27100 Pavia, Italy; 3 Department of Medical Sciences, University of Ferrara, Via Aldo Moro 8, 44124 Ferrara, Italy; 4 Geriatric Unit, Department of Medicine, University of Padova (UNIPD), Via Giustiniani 2, 35128 Padova, Italy; giuseppe.sergi@unipd.it; 5 Neuroscience Institute, Aging Branch, National Research Council (CNR), Viale Giuseppe Colombo 3, 35121 Padova, Italy; stefania.maggi@in.cnr.it (S.M.); marianna.noale@in.cnr.it (M.N.); 6 School of Agriculture and Food Science, University College Dublin, Belfield, D04 C1P1 Dublin, Ireland; lorraine.brennan@ucd.ie; 7 Division of Human Nutrition, Wageningen University, Box 17, 6700 AA Wageningen, The Netherlands; lisette.degroot@wur.nl; 8 Institute for Biomedicine of Aging, Friedrich-Alexander Universität of Erlangen-Nümberg, Kobergerstrasse 60, 90408 Nuremberg, Germany; dorothee.volkert@fau.de; 9 Centre for Public Health, Institute of Clinical Sciences, Queen’s University Belfast, First Floor Block A, Grosvenor Road, Belfast BT12 6BJ, UK; c.mcevoy@qub.ac.uk; 10 The Global Brain Institute, Trinity College Dublin, Ireland & University of California, 1651 4th St, 3rd Floor, San Francisco, CA 94158, USA

**Keywords:** retrospective data harmonization, pooled dataset, dietary data, observational studies, population-based studies

## Abstract

**Objectives:** The PROtein-enriched MEDiterranean diet to combat undernutrition and promote healthy neuroCOGnitive ageing in older adults (PROMED-COG) is a European project that investigates the role of nutritional status on neurocognitive ageing. This methodological paper describes the harmonization process of dietary data from four Italian observational studies (Pro.V.A., ILSA, BEST-FU, and NutBrain). **Methods:** Portion sizes and food frequency consumption within different food frequency questionnaires were retrospectively harmonized across the datasets on daily food frequency, initially analyzing raw data using the original codebook and establishing a uniform food categorization system. Individual foods were then aggregated into 27 common food groups. **Results:** The pooled cohort consisted of 9326 individuals (40–101 years, 52.4% female). BEST-FU recruited younger participants who were more often smokers and less physically active than those of the other studies. Dietary instruments varied across the studies differing in the number of items and time intervals assessed, but all collected dietary intake through face-to-face interviews with a common subset of items. The average daily intakes of the 27 food groups across studies varied, with BEST-FU participants generally consuming more fruits, vegetables, red meat, and fish than the other studies. **Conclusions:** Harmonization of dietary data presents challenges but allows for the integration of information from diverse studies, leading to a more robust and statistically powerful dataset. The study highlights the feasibility and benefits of data harmonization, despite inherent limitations, and sets the stage for future research into the effects of diet on cognitive health and aging.

## 1. Introduction

Over the past few decades, the pooling of datasets combining individual-level data from different studies has become increasingly common in epidemiological research [1,2,3,4,5]. Pooling different datasets has several advantages. First, it improves the comparability of research data collected in independent studies. Second, a larger sample size increases statistical power, which supports more robust conclusions. Third, the diversity of participants improves the ability to examine the effect modification by third factors (e.g., sex, socio-economic status, etc.). Fourth, using existing data provides a cost-effective approach that can also foster research collaboration [6,7]. In addition, unlike in study-level meta-analysis, joint analysis of individual-level data offers the possibility of re-using data in new ways by combining individual data from different studies. This could increase the diversity of samples and the robustness of statistical subgroup analyses (i.e., increase statistical efficiency and flexibility) [8].

Data harmonization, both prospective and retrospective, allows for integrating and aggregating data from multiple sources with common characteristics. Prospective harmonization occurs when investigators collaborate to establish guidelines for data collection, management, and pooling before studies begin. Retrospective harmonization is achieved by combining data from different studies after data collection, using domain expert knowledge to identify and translate study-specific variables into variables with comparable definitions and units [9].

The latter strategy has gained popularity in nutritional epidemiology in recent years [10,11,12,13,14,15,16,17,18] because it allows for a thorough, valid, and reliable investigation of the relationship between diet and diseases. Taking advantage of previously collected datasets, retrospective harmonization approaches could facilitate more effective use of previous research to improve the ability to answer complex questions on the role of diet in health that individual studies are not powered to address and to better inform current dietary guidelines and clinical practice for the population [19]. However, several methodological issues need to be recognized and faced in these harmonization approaches and subsequent pooled analyses. For instance, challenges concern differences in study objectives, design and setting, participant selection criteria, data collection procedures, dietary assessment methods, and dietary data quality, which result in heterogeneity between studies [9,20].

In this scenario, this article provides a detailed description of the variables retrieved from four Italian observational population-based studies included in the collaborative protein-enriched Mediterranean diet to combat undernutrition and promote healthy neurocognitive ageing in older adults (PROMED-COG) [21] Pooled Cohort Study. One of the main objectives of the project was to assess the impact of undernutrition on cognitive aging using epidemiological observational data. Undernutrition is a common condition in older adults, with a prevalence of up to 17% in the community and even higher in hospitalized or institutionalized individuals [22,23]. The relevance of undernutrition is due to its impact on several health-related outcomes, including quality of life, physical performance, frailty, and mortality [24,25,26,27]. Moreover, there is increasing evidence of a detrimental effect of undernutrition on cognitive function, although the heterogeneity of exposures considered in different studies makes it difficult to draw solid conclusions [28]. It is noteworthy that the PROMED-COG project evaluates not only undernutrition, but also the association between specific dietary habits such as adherence to the Mediterranean diet with cognitive decline and incident dementia.

From a theoretical perspective, understanding the methodology behind data pooling and harmonizing dietary data is crucial for advancing nutritional epidemiological research. It provides insights into how different datasets can be integrated to produce more comprehensive and accurate results. In practical terms, this study may represent a blueprint for researchers who want to combine data from different sources to improve the reliability of conclusions about diet and cognitive health.

To achieve these goals, the present study reports the step-by-step methodology that drives the harmonization process, on dietary data in particular, outlining the challenges encountered and the strategies used to overcome them.

## 2. Materials and Methods

### 2.1. General Description of the Study

The Collaborative PROMED-COG Pooled Cohort Study is being carried out as part of the PROMED-COG Project [21] (2021–2024), which receives funding from the European Horizon 2020 Joint Programming Initiative “a Healthy Diet for a Healthy Life” (JPI-HDHL) and the ERA-NET Cofund ERA-HDHL, specifically through the PREVNUT call for the development of targeted nutrition for prevention of undernutrition for older adults. PROMED-COG comprises five Work Packages (WPs) that bring together a multidisciplinary scientific team with a combination of expertise in epidemiology, gerontology, nutrition, biostatistics, metabolomics, and public health from the UK, Ireland, Italy, the Netherlands, and Germany, alongside external stakeholders.

### 2.2. Study Population

In particular, the first two work packages of the PROMED-COG project aim to use data from four independent, pre-existing, observational, population-based studies that are considered to be of adequate quality in terms of nutrition, exercise exposures, and neurocognitive outcomes, namely the Italian Longitudinal Study of Ageing (ILSA) [29,30], The Progetto Veneto Anziani (Pro.V.A) [31], the Italian Bollate Eye Study—Follow-up (BEST-FU) [32,33], and the Nutrition, Gut microbiota, and Brain Aging Study (NutBrain) [34]. The main characteristics of the four studies are briefly described below.

The Italian Longitudinal Study of Ageing (ILSA) is a population-based longitudinal study aiming to assess the prevalence and incidence rates of chronic conditions and associated risk factors among Italians aged 65–84 years in 1992–1993 (baseline), with active follow-ups in 1995–1996 and 2000–2001. A random sample of 5632 individuals was identified from the demographic lists of the registry office of eight municipalities in the North, Central, and South of Italy. All individuals aged 65–84 years who were free-living or institutionalized, residing in the study areas at the start of the prevalence study, and providing informed consent to participate were eligible. The survey was performed in two phases. The first phase, administered to all participants, included a personal interview on self-reported conditions, risk factors, and socio-demographic, behavioral, and health characteristics. Additionally, laboratory tests, physical examinations, and selected diagnostic tests were performed. Only participants who screened positive in the first phase were considered in the second phase, which consisted of the clinical confirmation by a specialist of suspected cases of diabetes and cardiovascular and neurological conditions.

The Progetto Veneto Anziani (Pro.V.A) is a longitudinal study examining determinants of disability in an age- and a sex-stratified random sample of 3099 adults aged ≥65 years living in the Veneto region, Northern Italy, who provided their consent to participate in the study. The sampling procedures involved all individuals aged 65 and older living in the cities of Camposampiero (PD) and Rovigo, with no exclusion criteria. The baseline assessment was performed in 1995–1997, and active follow-ups in 1999–2000 and 2002–2004. A passive follow-up using regional health registers to derive hospitalization and mortality data was recently performed until 2018. All participants underwent a comprehensive evaluation at the local involved hospital or home (for those who were not able to show up to the research site) with trained nurses/physicians who assessed socio-demographic characteristics, cognitive performance, dietary intake, disease symptoms, and functional status. Moreover, a comprehensive physical examination was performed by a nurse and physician, and blood samples were collected. A subsample of participants free from dementia also underwent brain Magnetic Resonance Imaging (MRI).

The Italian Bollate Eye Study—Follow-up (BEST-FU) is a cohort study involving 1693 dementia-free community-dwelling individuals from the Lombardy region, Northern Italy. All free-living individuals aged 40 years and older living in the study area where the study was conducted who provided informed consent to participate were eligible. The study included a baseline evaluation in 1992–1993, and participants were monitored for over 20 years using electronic health records. At the baseline, individuals underwent a clinical examination to assess their socio-demographic, lifestyle, and medical history. A detailed assessment of their nutritional status was carried out, along with blood pressure measurement, and fasting blood was taken for biochemical analysis.

The Nutrition, Gut microbiota, and Brain Aging Study (NutBrain) is a cross-sectional, population-based cohort of 804 adults living in the Lombardy region. All free-living individuals aged 65 years or older who lived in the study area where the study was conducted and provided informed consent to participate were eligible. During the screening phase, individuals underwent cognitive assessment. Based on their cognitive performance, a sub-sample of 254 participants underwent a complete clinical evaluation, including neurological examination and structural and functional MRI brain scanning. Samples of stool and blood were also collected.

The data from all studies considered were anonymized.

In the PROMED-COG Pooled Cohort Study, the longitudinal association of nutritional status and exercise on cognitive outcomes is investigated using the ILSA, Pro.V.A, and BEST-FU studies. The Pro.V.A and NutBrain studies are employed to provide a mechanistic understanding of the relationship between diet and neurocognition through a cross-sectional design.

### 2.3. Nutritional Status and Dietary Data

Nutritional status was derived based on several variables measured in each dataset, as described below.

In the ILSA study, height was measured by a stadiometer (Salus) at head level to the nearest centimeter with the subject standing barefoot, with feet together. Body weight was measured on a balance beam platform scale (Salus, Milan, Italy) to the nearest 0.1 kg, with the subject lightly dressed. In the Pro.V.A study, body weight and height were measured with individuals wearing light indoor clothing and no shoes through balance scales accurate to the nearest 0.1 kg and stadiometer to the nearest 0.01 m. The waist circumference measure was taken in the middle between the lowest rib and the iliac crest (with individuals in the standing position). In the BEST-FU study, the participants were weighed while wearing only their underclothes, and their height was measured while standing fully erect without shoes. In the NutBrain study, a qualified dietician measured the body weight, height, and waist and mid-upper arm circumferences of participants. Body weight (in kilograms to the nearest 0.5) was assessed using bioelectrical impedance analysis (BIA) through a homologated leveled-platform electronic scale (Tanita SC-240MA, Hoogoorddreef 56E, 1101 BE Amsterdam, The Netherlands), with participants wearing light clothing and no shoes. Height (in centimeters to the nearest 0.5) was measured using a portable wall-mounting system, with participants standing shoeless (SECA 213).

Body Mass Index (BMI) was calculated using the standard formula (weight/height^2^).

Dietary habits collection in the four cohorts is reported below.

In the ILSA study, a trained interviewer collected dietary data using a 49-item semi-food frequency questionnaire (SFFQ). The participants reported the frequency of their average intake for each food consumed during the previous week. Alcohol consumption was measured in liters consumed every day and then converted into daily glasses. Standard portion sizes were used to measure food consumption.

In the Pro.V.A. study, an interviewer trained for the task administered a 52-item FFQ. The participants were asked to report how often they consumed each food on average during a usual week in the last 2–3 months. Standard portion size was used to determine the amount of food consumed. For the Pro.V.A. study, only in the case of some dietary components having more than 20% missing values, the median consumptions by sex and age were imputed from the ILSA study (which was comparable to the Pro.V.A. study in terms of baseline year, study population, and design).

In the BEST-FU study, a quantitative FFQ was used to assess dietary habits in the year before recruitment. The FFQ was adapted from Willett’s questionnaire in the Nurses’ Health Study [35] and administered by a trained interviewer. It consisted of a list of 158 foods items, and participants were asked to report how often they consumed each item over the past year using a seven-frequency scale ranging from never to 4–5 times per day. The amount of food consumed was determined by selecting a picture of a food portion.

In the NutBrain study, a 102-item SFFQ (adapted from the validated questionnaire by Willet in the Nurses’ Health Study [35]) was used to gather dietary habits within the previous year. Respondents were requested to indicate the frequency of consuming a standard portion of the given food using a nine-category frequency scale. The options ranged from never/seldom (less than once per month) to 4–5 times a day. Color pictures illustrating the serving size of each food item were presented to aid comprehension of standard portion sizes. Details regarding the four cohorts and the dietary data gathering are reported in Table 1.

### 2.4. Step-by-Step Retrospective Harmonization Procedure

**Figure 1:**
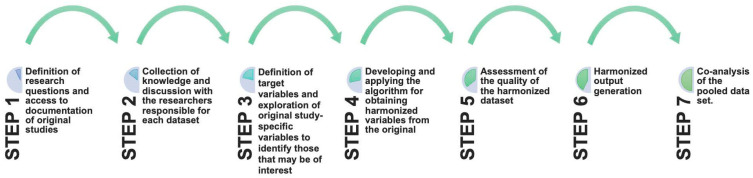
The process adopted in the PROMED-COG project to establish and harmonize variables from the four datasets.

Retrospective data harmonization was performed according to existing guidelines [7]. The harmonization team consisted of epidemiologists, statisticians, and nutritionists. The process adopted to establish and harmonize the variables can be divided into seven steps as previously described [37]; the framework is displayed in Figure 1.

### 2.5. Evaluation of Information on Potentially Relevant Nondietary Covariates

According to the study aims, harmonized nondietary variables were also created for each dataset, including general information about the study, socio-demographic characteristics, health status variables, neurocognitive outcomes, and other lifestyles. The complete list of variables is displayed in Supplementary Table S1.

### 2.6. Statistical Analysis

Harmonized continuous variables are summarized as means and SD or median and interquartile ranges (IQRs) by the cohort study. Meanwhile, harmonized categorical variables are presented as counts and percentages. Normal distributions of continuous variables are tested using the Shapiro–Wilk test. To assess the existence of differences in the distribution of harmonized variables across the cohort study and by sex, Chi-square or Fisher exact tests for categorical variables and generalized linear models after testing for homoscedasticity (Levene test) or Wilcoxon rank-sum test for continuous variables were considered. All the analyses were performed using SAS release 9.4 (SAS Institute Inc., Cary, NC, USA), Stata 15.0 version (StataCorp LP, College Station, TX, USA) and SPSS Statistics version 25.0 (IBM Corp., Armonk, NY, USA) statistical packages. Two-tail *p*-values < 0.05 were considered statistically significant.

### 2.7. Data Management and Confidentiality

The study protocols were implemented in compliance with the guidelines outlined in the Declaration of Helsinki. The responsible Ethics Committees approved all procedures; for the ILSA study, it was approved by the institutional review board of the eight participating municipalities listed in [29], while for the Pro.V.A. study, it was approved by the Ethics Committee of the University of Padova and of the number 15 and 18 Local Health Units of the Veneto Region. The BEST-FU study was approved by the Ethics Review Board of the CNR of Segrate (MI). The NutBrain study was approved by the Medical Ethics Committee of Pavia (approval Prot. 20180036036, 20 April 2018, amendment Prot. 20190045757, 21 May 2019). For original studies conducted by ILSA and Pro.V.A., written informed consent was obtained from all participants. For the BEST-FU study, verbal informed consent was witnessed and formally recorded by all participants. In the NutBrain study, all participants provided formal written informed consent.

Data were handled and stored in accordance with the European General Data Protection Regulation (EU) 2016/679 (GDPR) (https://gdprinfo.eu/, accessed on 1 September 2021). The file server was firewalled within the National Research Council (CNR) intranet. To ensure privacy and security, access to the database required a password granted only to the server administrator. Data transfer was protected using an encrypting/decrypting policy and password protection. In the final dataset, a unique key was assigned to each subject to guarantee anonymity. Personal data were considered confidential and removed before the exportation process. Data security was guaranteed through automatic backups. Anonymized original data in Excel, SPSS, and SAS formats were provided by the responsible of each study, along with definition and formatting information for each variable.

## 3. Results

### 3.1. Comparison of Dietary Variables

The instruments used to collect dietary data were the FFQ in the BEST-FU and Pro.V.A. studies and the SFFQ in the ILSA and NutBrain studies, with some differences in the number of items: 158 in BEST-FU, 52 in PRO.V.A., 49 in ILSA, and 102 in NutBrain. There was also a difference in the time interval asked about in the four FFQs, with the BEST-FU and NutBrain questionnaires asking about intake during the previous 12 months, whereas the Pro.V.A. and ILSA asked about dietary intake during, respectively, a usual week in the previous 2–3 months, and the previous week. Other similarities and differences between the four FFQs are highlighted in Table 1. In all the FFQs, dietary intake was collected through face-to-face interviews. The number of items common to all studies was 27 for BEST-FU, 25 for Pro.V.A., 24 for ILSA, and 27 for NutBrain.

### 3.2. Harmonization of the Dietary Data and Food Group Intake

As the FFQs included a variety of foods, with questions about different response categories and quantities asked in different ways, conversion procedures were used to obtain harmonized data on the daily frequency of consumption of each food.

The research team first analyzed the dietary data using the original data codebook and descriptive statistics. Second, they converted the raw frequency variables in all food questionnaires to the amount consumed in grams per day for each food item and harmonized the food portion sizes based on Italian guidelines [38], and a uniform food categorization system was established. Third, nutrient and energy intakes were calculated using the Italian food composition databases for epidemiological studies in Italy [36].

Fourth, despite some different FFQ items in each dataset, the individual food items were partially comparable and aggregated into 27 well-known higher-order food groups included in each dataset and defined in such a way that the classifications were as similar as possible across the four cohorts, as shown in Table 2. The 27 food groups were fruit, vegetables, potatoes, red meat, white meat, cured meat, legumes, fish and sea products, tuna in oil, dairy products, yogurt, cheese, cereals, bread and substitutes, stuffed pasta, egg, dried fruit, sweets and snacks, fats, oils, mineral water, unsweetened beverages, sugary beverages, spirits, sugar, salt and spices, and dietetic products. Some food groups were not available for all cohorts, e.g., “stuffed pasta” and “salt and types” were not available for ILSA, while “tuna in oil” and “mineral water” were not available for Pro.V.A.

**Figure 2:**
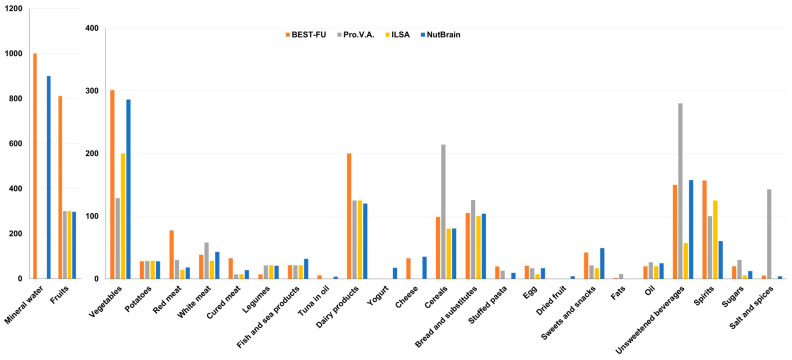
Food group intake distribution (median g/day) by study.

Intakes (g/day) of 27 major food groups across studies are reported in Supplementary Table S2, Figure 2. For instance, in the pooled sample, the average daily intake was 386 g for fruits and 200 g for vegetables. There was some variation in fruit and vegetable consumption across the studies, with BEST-FU participants consuming more compared to those in the Pro.V.A., ILSA, and NutBrain studies. Additionally, BEST-FU participants had higher intake of food groups such as red meat, fish, dairy products, stuffed pasta, sweets and snacks, and spirits compared to participants in other studies.

Supplementary Table S3 shows that the following food groups were statistically significantly lower in females compared to males: potatoes, red and cured meats, legumes, fish and seafood and tuna, bread and substitutes and filled pasta, fats, water, and spirits. Conversely, the following food groups were more common in females than males: eggs, sweets and snacks, and unsweetened beverages.

### 3.3. Comparison of the Nondietary Variables of the Four Cohorts

The pooled cohort consists of 9326 individuals with data collection years ranging from 1992 to 2023. Individuals from the original datasets living in long-term care facilities and those with all missing variables required for harmonization were excluded. The main characteristics of the cohort, pooled and by original study, are presented in Table 3, and marked differences by original studies were found.

The mean age of the participants at baseline was 72.4 ± 9.5 years (range 42–101); the age distribution was similar between the studies, except for the BEST-FU study, which recruited younger participants compared to Pro.V.A, ILSA, and NutBrain (59.6 ± 7.9 years for BEST-FU, 76.0 ± 7.7 years for Pro.V.A., 74.7 ± 5.7 years for ILSA, 75.6 ± 6.3 years for NutBrain).

The sex distribution of participants was generally similar across studies. Heterogeneity among the original studies was found for marital status, educational level, occupation, and socio-economic status. Compared to the other three studies (BEST-FU, Pro.V.A., and ILSA), the NutBrain cohort was more likely to be separated or divorced, highly educated, white collar, and of higher socio-economic status. Regarding nutritional status, the mean BMI of the whole sample was 27.1 ± 4.4, with no significant differences between studies, the median energy intake was 2767 (2518–3254) in the whole sample; compared to the other three studies, energy intake was lower in the NutBrain cohort. In terms of lifestyle and health status, participants in the BEST-FU study were more likely to be current smokers and less physically active. Data on mobility limitations were not available in the BEST-FU study, while the ILSA study lacked a complete baseline assessment of physical activity levels.

Supplementary Table S4 shows the non-dietary characteristics of the cohorts by sex. Compared to males, females were significantly less educated, housewives, widowed, and of lower socio-economic status. They had a higher BMI, consumed fewer daily calories, were more likely to be non-smokers, to take more daily medication, to have more mobility limitations, and to be less physically active.

## 4. Discussion

The Collaborative PROMED-COG Pooled Cohort Study is part of a larger European project aimed at studying the role of undernutrition on neurocognitive aging. The present manuscript outlines a methodological approach to harmonize and pool dietary data from four Italian observational studies, resulting in a large population-based sample of more than 9000 participants from young adulthood to old age. Although many previous studies have applied harmonization to previously collected dietary data from different datasets [10,11,12,13,14,15,16,17,18,19,20], to our knowledge, this is the first study that has pooled dietary variables to develop a large Italian database to analyze dietary exposure in relation to cognition.

### Methodological Considerations

Harmonization of dietary data presented many challenges. Several approaches were used to overcome these, including the revision of food-level data, the definition of comparable dietary and non-dietary variables, and the development of a food grouping system that was implemented across the four observational studies.

Aggregating data from these four studies has several methodological advantages.

They all have similar characteristics in terms of the geographical area from which participants were recruited (Italy), the method of data collection (by trained interviewers), and the population-based, community-dwelling setting. This facilitates comparison between studies and leads to more consistent and reliable results. We attempted to harmonize exposures, outcomes, and confounders to remove potential sources of heterogeneity and lack of comparability.

With regard to dietary exposures, the pooled data set allowed us to examine associations over a wide range of dietary exposures (a priori and a posteriori dietary patterns, macro- and micronutrient intakes, food items, food groups) with greater precision than in the individual studies, because the larger sample size facilitates statistical analyses that would have been less feasible using either data set alone.

Another aspect is that we used the same Italian food composition database to derive energy intakes and macro- and micronutrient intakes in all studies, which increases the comparability between studies. The use of portion sizes should be less problematic when comparing intake estimates because the same source material was used to determine standard portion sizes.

In addition, despite some unique foods within each FFQ, the foods were comparable and aggregated into 27 common higher-order food groups (e.g., fruits, legumes, etc.) that were identified and constructed to (i) allow comparisons between the four cohorts, (ii) allow the analysis of specific types of foods, and (iii) allow groups to be categorized into more general categories to develop scores and patterns without loss of information [13]. Furthermore, the ILSA, Pro. V.A, and BEST-FU studies are prospective in their design; therefore, nutritional status and lifestyle were assessed before the development of outcomes, limiting recall and selection bias, and reverse causation.

More generally, the retrospective harmonization and pooling of data from these four studies increased the sample size, thereby increasing the statistical power and robustness of the conclusions drawn, allowing us to examine how different factors, such as gender or socioeconomic status, modify the associations under investigation and to re-use data to increase the diversity of samples and the robustness of statistical subgroup analyses.

However, the Collaborative PROMED-COG Pooled Cohort Study has some limitations. First, being designed retrospectively, it faces inevitable heterogeneity in study design and execution, including differences in objectives, sampling frames, recruitment procedures, data collection periods, and selected questionnaires and scales, making harmonization challenging. The studies involved populations of different ages (ILSA, Pro.V.A., and NutBrain focused on individuals aged 65 and older, whereas BEST included middle-aged participants), educational and socioeconomic levels (lower in Pro.V.A. and ILSA compared to higher in BEST-FU and NutBrain), and follow-up data availability (active in ILSA and Pro.V.A, passive in BEST-FU, and unavailable in NutBrain). To address these differences, in future analyses, we will consider socio-demographic, economic, and cohort-related factors, which can act as confounders or effect modifiers in the association between nutritional status and cognitive health.

Second, there is a potential for cohort bias, as Pro.V.A, ILSA, and BEST-FU were conducted almost twenty years before NutBrain. Although analytical models can control for cohort membership, the unique conditions, environments, and resources of each study population may influence exposure patterns and health outcomes.

Third, the dietary assessment methods varied across studies in terms of the number of items (158 in BEST-FU, 52 in PRO.V.A., 49 in ILSA, and 102 in NutBrain), data collection periods (1992–1997 for ILSA, Pro.V.A., and BEST-FU, and 2019–2023 for NutBrain), and reporting intervals (last week for ILSA, a typical week in the last 2–3 months for Pro.V.A., and the last 12 months for BEST-FU and NutBrain). These differences can introduce artefactual differences in estimated intakes, affecting data comparability [12].

Fourth, the diet was collected in all the studies using a single administration of dietary questionnaires, so recall bias and measurement error are recognised limitations of these instruments.

Fifth, despite comparable dietary assessment methodologies, significant differences in dietary habits were observed between the studies, leading to greater variation in dietary exposure in combined analyses. However, combined analyses can enhance the range of exposures and statistical power when examining risks for rare or relatively common disease outcomes [13,17].

Sixth, variation in dietary data quality, including differences in frequency of consumption and portion sizes, may affect the accuracy of harmonized data [9].

Finally, harmonizing data requires considerable expertise to identify and translate study-specific variables into comparable definitions and units, which can be resource-intensive and time-consuming [39].

These benefits and limitations highlight the importance of careful planning and implementation of dietary data harmonization to ensure the reliability and validity of research findings [17].

## 5. Conclusions

In summary, despite some limitations, pooling data on dietary exposures from different population-based studies is a valuable resource for investigating the health effects of relatively rare dietary exposures and assessing rare disease outcomes. In particular, in the next steps of the PROMED-COG project, we will use the harmonized dataset to i) provide estimates of the burden of undernutrition on cognitive decline and dementia incidence and to identify determinants of weight trajectories and undernutrition in the population; ii) assess the potentially additive effects of nutritional status and lifestyle, including exercise, on longitudinal neurocognitive outcomes; and iii) elucidate potential mechanisms of dietary behaviors on brain structures. However, the harmonization process described in this paper may also be applicable and feasible in other contexts, representing a way to advance future nutritional epidemiological research.

## Figures and Tables

**Table 1:** Characteristics of the studies included in the PROMED-COG Pooled Cohort Study, Italy, 1992–2023.

Study	Study Site	Baseline Recruitment	Study Population	Study Design	Dietary Assessment Methods						
					Type	No. of Items	No. of Common Items	Time Frame	Components Measured	Administration	Nutrient Calculation
Italian Bollate Eye Study Follow-up—BEST-FU [32,33]	Lombardy region, North of Italy	1992–1993	1604, age range 40–74, 50.3% females	Prospective, passive follow-up (20 y)	FFQ	158	27	Past year	Daily frequency and portion size (grams)	Face-to-face interview	Italian Food Composition Databases for Epidemiological Studies in Italy [36]
Progetto Veneto Anziani—Pro.V.A. [31]	Veneto region, North of Italy	1995–1997	3099, age range 65–101, 59.1% females	Prospective, active and passive follow-up (7 + 16 y)	FFQ	52	25	Past week	Daily frequency and portion size (grams)	Face-to-face interview	Italian Food Composition Databases for Epidemiological Studies in Italy [36]
Italian Longitudinal Study of Ageing—ILSA [29,30]	North, Central and South of Italy	1992–1993	5632, age range 65–85, 48.6% females	Prospective, active follow-up (8 y)	SFFQ	49	24	Past week	Daily frequency of standard portion size (grams)	Face-to-face interview	Italian Food Composition Databases for Epidemiological Studies in Italy [36]
Nutrition, Gut microbiota, and Brain Aging Study—NutBrain [34]	Lombardy region, North of Italy	2019–2023	254, age range 65–94, 59.1% females	Cross-sectional	SFFQ	102	27	Past year	Daily frequency of standard portion size (grams)	Face-to-face interview	Italian Food Composition Databases for Epidemiological Studies in Italy [36]

**Table 2:** Single food items and common higher-order food groups considered in each dietary database.

		BEST-FU	NUTBRAIN	PRO.V.A.	ILSA	
Food Groups	Common Foods	Food Items in Defined Food Groups				Frequency of Consumption (Median no. Servings/Week)
Cereals	Pasta Rice Semolina	Rice Maccheroni Pasta Fresh egg pasta Semolina Pasta prepared with broth Beans and pasta Polenta Orecchiette	Pasta Rice Fresh egg pasta Pasta or rice in broth (semolina, pancotto) Pasta and beans/lentils/ peas Polenta	Pasta Rice Broth soup Sliced polenta Semolina Barley Cereals/bran	Pasta/rice/semolina Fresh egg pasta	BEST-FU: 10 NUTBRAIN: 7 Pro.V.A.: 11 ILSA: 7
Bread and panel substitutes	Bread Crackers Breadsticks	Bread Focaccia Pizza Crackers Melba toast Breadsticks	Bread Pizza Crackers Rusks Breadsticks	White bread Whole grain bread Breadsticks/ crackers Rusks Salty piece/pizza Panbiscotto Pizza	Crackers/rusks/ breadsticks Bread	BEST-FU: 14 NUTBRAIN: 13 Pro.V.A.: 9 ILSA: 14
Stuffed pasta	N.A.	Rice salad Tortellini Cannelloni Lasagne Tortelloni	Ravioli/tortellini/ tortelli Lasagna/ cannelloni	Stuffed pasta	n.a.	BEST-FU: 1 NUTBRAIN: 0 Pro.V.A.: 0 ILSA: -
Potatoes	Potatoes	Boiled potatoes Mashed potatoes French fries Soft pasta with potatoes	Potato gnocchi Potatoes	Potatoes	Potatoes	BEST-FU: 1 NUTBRAIN: 0 Pro.V.A.: 1 ILSA: 1
Egg	Egg	Whole egg Omelet	Whole egg/omelet	Egg	Egg	BEST-FU: 2 NUTBRAIN: 2 Pro.V.A.: 2 ILSA: 1
Red meat	Red meat	Canned beef Lean grilled beef Horse Hamburger Cutlet Pork chop Meatball Stew with potatoes Stew with peas Beef stew Veal with tuna sauce Lamb or kid Entrails	Slices of beef/veal/horse (meatballs, Swiss, schnitzel) Grilled pork chop Lamb or kid Offal (liver, kidney) Meat sauce (ragù)	Meat sauce Red meat Meat homogenized	Red meats (veal, beef, pork, horse) Liver/heart/ kidney/tongue Canned meat	BEST-FU: 5 NUTBRAIN: 1 Pro.V.A.: 2 ILSA: 2
White meat	White meat	Roast turkey Rabbit Chicken leg quarters Chicken breast Game bird	White meat (turkey, chicken, rabbit)	White meat	White meats (chicken, turkey, rabbit) Game (hare, pheasant)	BEST-FU: 2 NUTBRAIN: 3 Pro.V.A.: 4 ILSA: 2
Cured meat	Ham Salami	Cured ham Sausages Coppa Bacon Ham Bresaola Speck Salami	Raw ham/speck Fresh sausage Ham cooked Bresaola Other cold cuts (salami, cotechino/wurstel, bacon, mortadella, coppa)	Ham without fat Ham with fat Salami/ mortadella/ soppressa	Ham/salami/ mortadella	BEST-FU: 4 NUTBRAIN: 1 Pro.V.A.: 1 ILSA: 1
Legumes	Legumes	Beans Peas	Legumes (beans, chickpeas, peas, broad beans, lentils)	Legumes	Fresh legumes (peas, beans) Dried legumes (chickpeas, lentils)	BEST-FU: 0 NUTBRAIN: 0 Pro.V.A.: 0 ILSA: 1
Fish and sea products	Fish	Dover sole Mackerel Anchovies Trout Hake Eel Crustaceans	Lean fish (sole, trout, cod, hake, sea bream, sea bass) Fatty fish (salmon, tuna, swordfish) Oily fish (anchovy, sardines, mackerel, anchovies) Crustaceans/ mollusks (prawns, scampi, mussels, clams)	Fish	Fish such as eel, mackerel, fresh tuna, oily fish, hake, swordfish, salmon Mollusks (clams, mussels, oysters) Crustaceans (shrimp, lobster)	BEST-FU: 1 NUTBRAIN: 1 Pro.V.A.: 1 ILSA: 1
Tuna in oil	N.A.	Tuna canned in oil	Tuna in oil	N.A.	Canned tuna	BEST-FU: 0 NUTBRAIN: 0 Pro.V.A.: - ILSA: 0
Dairy products	Milk	Whole milk Semi-skimmed milk Skimmed milk	Whole milk/cappuccino/ coffee-milk Milk semi-skimmed Skimmed milk	Milk Cappuccino	Whole milk Milk semi-skimmed Skimmed milk	BEST-FU: 11 NUTBRAIN: 6 Pro.V.A.: 14 ILSA: 7
Yogurt	Yogurt	Yogurt with fruit Skimmed yogurt Whole yogurt	Low-fat yogurt Whole yogurt	Yogurt	Yogurt	BEST-FU: 0 NUTBRAIN: 0 Pro.V.A.: 2 ILSA: 0
Cheese	Parmesan Fresh cheese Seasoned cheese	Processed cheese Crescenza Parmesan Pecorino Ricotta Emmenthal Mozzarella Provola chili Provola sweet	Crescenza/ spreadable cheese Grana/parmesan/pecorin Ricotta/cottage cheese Mozzarella/provola/scamorza Other cheeses (gruyere/brie/ gorgonzola/taleggio)	Parmesan Fresh cheese Seasoned cheese	Cheese like mascarpone, gorgonzola Cheese such as Bel Paese, Emmenthal, mozzarella, parmesan, stracchino, taleggio, etc. Cheese such as ricotta	BEST-FU: 3 NUTBRAIN: 3 Pro.V.A.: 2 ILSA: 3
Vegetables	Fresh (or frozen) vegetables	Lettuce Tomatoes Raw fennel Celery Green beans Asparagus Cucumber Cooked fennel Cooked carrots Cooked courgettes Swiss chard Raw carrots Raw peppers Artichokes Cauliflower and broccoli Onions Mushrooms Aubergine Spinach Cabbage Tomato canned Tomato puree Mixed vegetables and pulses	Raw leafy vegetables (lettuce, lamb’s lettuce, radicchio, chicory) Tomatoes Fennel Green beans Asparagus Cucumbers Courgettes Chard Carrots Peppers Artichokes Cauliflower/broccoli/cabbage/cabbage Onion (also in sauté) Mushrooms Aubergines Spinach Minestrone Tomato sauce/puree	Tomato sauce Vegetable/salad Minestrone Cooked vegetables	Fresh (or frozen) vegetables	BEST-FU: 12 NUTBRAIN: 14 Pro.V.A.: 11 ILSA: 7
Fruits	Fresh fruits	Apricot Orange Banana Cherry Watermelon Fig Lemon Strawberry Clementines and tangerines Pear Apple Peach Grapefruit KiwiPlum Grape Fruit juice	Apricot Orange/orange juice (no sugar) Banana Cherry Watermelon/melon Strawberry/raspberry/blueberry Clementines/ tangerines Pear Apple Peach Kiwi Plum Grape	Raw fruit Cooked fruit Fresh fruit Homogenized fruit Juice (no sugar) Smoothie	Fresh fruits (orange, apple, banana, pear, peach, etc.)	BEST-FU: 37 NUTBRAIN: 13 Pro.V.A.: 21 ILSA: 14
Dried fruit	Dried fruit	Dried fruit	Dried fruit (walnuts, hazelnuts, peanuts)	Dried fruits	Dried fruits (peanuts, walnuts, almonds, etc.)	BEST-FU: 0 NUTBRAIN: 0 Pro.V.A.: 7 ILSA: 0
Sweets and snacks	Biscuits Chocolate Ice cream	Biscuits Little cake commercially prepared Chocolate Sponge cake Cake Milk ice cream Fruit ice cream	Common cookies (dry, shortbread, milk biscuits, etc.) Brioche/pastries Cake (daisy cake, chocolate cake) Chocolate Cream ice cream Fruit ice cream Potato chips in bag	Sweet smoothie * Biscuits Brioche Piece of cake Ice cream Chocolates Salty smoothie	Biscuits Desserts with whipped cream or cream/ice cream Dry desserts (tarts, dry pastries) Chocolate	BEST-FU: 6 NUTBRAIN: 9 Pro.V.A.: 4 ILSA: 2
Fats	Butter Margarine	Butter Margarine Lard Fatback pork Mayonnaise Mascarpone	Butter Margarine Lard/Fatback pork	Margarine Butter Cooking cream	Butter Margarine Mayonnaise	BEST-FU: 0 NUTBRAIN: 0 Pro.V.A.: 4 ILSA: 0
Oil	Seed oil Olive oil	Seed oil Olive oil	Seed oil Olive oil	Seed oil Olive oil	Seed oil Olive oil	BEST-FU: 14 NUTBRAIN: 13 Pro.V.A.: 14 ILSA: 14
Mineral water	N.A.	Mineral water	Water	N.A.	N.A.	BEST-FU: 35 NUTBRAIN: 31 Pro.V.A.: - ILSA: -
Unsweetened drinks	Coffee Tea	Coffee Tea	Coffee Tea/herbal teas	Coffee Tea Chamomile/herbal tea Decaffeinated coffee	Coffee (excluding barley or decaffeinated coffee) Tea	BEST-FU: 17 NUTBRAIN: 17 Pro.V.A.: 13 ILSA: 7
Sugary drinks	Fruit juice	Beverages with gas Beverages without gas Fruit juice canned	Sweet drinks (carbonated and sweetener) Fruit juices	Fruit juice	Carbonated drinks (excluding water) Fruit juices	BEST-FU: 0 NUTBRAIN: 0 Pro.V.A.: 4 ILSA: 0
Spirits	Spirits Dessert wine Beer Wine	Spirits Dessert wine Fortified wine/cherry Beer Red wine White wine Rosè wine	Beer Red wine White wine Bitters or liqueurs (grappa, cognac, whiskey, digestives, or sweet liqueurs)	Spirits, dessert wine, cherry, beer, wine, alcohol	Spirits, dessert wine, cherry, beer, wine (red, white and rose)	BEST-FU: 7 NUTBRAIN: 2 Pro.V.A.: 4 ILSA: 5
Sugars	Candies Jam Sugar Honey	Candies Jam apricot Jam cherry Jam plumb Jam peach Other jam Sugar Honey	Candies Sugar Honey Jam	Sugar Jam/honey Candies	Sugar Jam Honey Candies	BEST-FU: 21 NUTBRAIN: 7 Pro.V.A.: 31 ILSA: 8
Salt and spices	N.A.	Salt Pepper Chili pepper	Salt	Broth/stock cube Other (lecithin, seasonings)	N.A.	BEST-FU: 9 NUTBRAIN: 3 Pro.V.A.: 7 ILSA: -
Dietetic products	Sweetener	Saccharin	Sweeteners	Sweetener	Sugar-free sweets or cookies Sugar-free drinks Artificial sweeteners (Dietor, saccharin)	BEST-FU: 0 NUTBRAIN: 0 Pro.V.A.: 0 ILSA: 0

N.A.: not available; no: number. * A sweet smoothie can include milk, fruit, egg, sugar; a salty smoothie can contain broth, meat, potatoes, parmesan cheese.

**Table 3:** Baseline characteristics by study.

	Overall Cohort (n = 9016)	BEST-FU (n = 1353)	Pro.V.A. (n = 2981)	ILSA (n = 4428)	NutBrain (n = 254)	*p*-Value
Baseline (year)	1991–2023	1991–1995	1995–1998	1992–1993	2023	
Socio-demographic variables						
Age, mean ± SD	72.4 ± 9.5	56.8 ± 8.1	76.0 ± 7.7	74.5 ± 5.7	75.6 ± 6.3	<0.0001
Sex, females, n (%)	4745 (52.6)	681 (50.3)	1762 (59.1)	2152 (48.6)	150 (59.1)	<0.0001
Education, n (%)						<0.0001
Primary school or less	6564 (73.3)	776 (57.4)	2615 (88.0)	3129 (71.5)	44 (17.2)	
Middle school	1256 (14.0)	420 (31.0)	212 (7.1)	551 (12.6)	72 (28.4)	
High school	768 (8.6)	131 (9.7)	96 (3.2)	442 (10.1)	99 (39.0)	
University or higher	369 (4.1)	26 (1.9)	49 (1.7)	255 (5.8)	39 (15.4)	
Work done for most of the time, n (%)						<0.0001
Housewife	1323 (15.1)	98 (7.2)	357 (12.1)	860 (20.3)	8 (3.2)	
Blue collar	4758 (54.2)	722 (53.4)	1794 (60.9)	2126 (50.2)	116 (45.6)	
White collar	2703 (30.8)	533 (39.4)	794 (27.0)	1246 (29.4)	130 (51.2)	
Marital status, n (%)						<0.0001
Single or never married	599 (6.6)	47 (3.5)	227 (7.6)	312 (7.1)	13 (5.1)	
Married or cohabiting	5475 (60.8)	1151 (85.0)	1536 (51.5)	2630 (59.5)	158 (62.2)	
Separated or divorced	104 (1.2)	23 (1.7)	16 (0.5)	47 (1.1)	18 (7.1)	
Widowed	2826 (31.4)	132 (9.8)	1201 (40.3)	1428 (32.3)	65 (25.6)	
Socioeconomic status, n (%)						<0.0001
Low	6123 (68.0)	816 (60.3)	2171 (72.9)	3049 (69.0)	87 (34.2)	
Medium	1922 (21.4)	384 (28.4)	679 (22.8)	793 (18.0)	66 (26.0)	
High	959 (10.7)	153 (11.3)	130 (4.4)	575 (13.0)	101 (39.8)	
Nutritional status						
Body Mass Index, kg/m^2^, mean ± SD	27.1 ± 4.4	27.0 ± 4.1	27.6 ± 4.6	26.8 ± 4.4	27.1 ± 4.2	<0.0001
Energy intake, kcal, median (Q1, Q3)	2767 (2518, 3254)	2841 (2194, 3586)	2686 (2652, 2863)	2786 (2258, 3175)	1937 (1648, 2255)	<0.0001
Lifestyle and Health status variables						
Smoking status, n (%)						<0.0001
Current smoker	1590 (17.7)	698 (51.6)	264 (8.9)	609 (13.8)	19 (7.5)	
Former smoker	2797 (31.1)	305 (22.5)	892 (29.9)	1510 (34.2)	90 (35.4)	
Never smoker	4614 (51.3)	350 (25.9)	1824 (61.2)	2295 (52.0)	145 (57.1)	
Number of medications ≥ 5, n (%)	1473 (17.3)	178 (13.2)	644 (26.2)	593 (14.5)	58 (22.8)	<0.0001
Mobility limitations, cannot walk, n (%)	328 (5.2)	N.A.	243 (8.2)	81 (2.7)	4 (1.6)	<0.0001
Physical activity ≥ 4 h/week, n (%)	814 (17.9)	70 (5.4)	689 (23.1)	N.A.	55 (21.7)	<0.0001

Q1, Quartile 1; Q3, Quartile 3; SD, Standard Deviation; N.A., not available.

## Data Availability

The data presented in this study are available on request from the corresponding author.

## Supplementary Materials

The following supporting information can be downloaded at: https://www.mdpi.com/article/10.3390/nu16223917/s1, Supplementary Table S1: Harmonized nondietary variables created for each domain; Supplementary Table S2: Food group intake distribution (g/day) by study; Supplementary Table S3. Food group intake distribution (g/day) by sex; Supplementary Table S4. Baseline characteristics by sex.
