# Supplementary material for: Harmonizing Dietary Exposure of Adult and Older Individuals: A Methodological Work of the Collaborative PROMED-COG Pooled Cohorts Study
Source: Nutrients. 2024 Nov 16;16(22):3917. doi: 10.3390/nu16223917 (PMC11597225; doi:10.3390/nu16223917)
Supplement: Supplementary file 1 [file nutrients-16-03917-s001.zip › nutrients-3252583-supplementary.pdf]

Supplementary Table 1. Harmonised nondietary variables created for each domain

**Domains**	**Harmonised variables created**
General information	Study ID and participants' ID; date of the interviews (baseline, first follow-up, second follow-up).
Socio-demographic characteristics	Sex, birth date, education (highest level of education achieved), work done most of the time (categorized into blue collars, white collars, housewives), and marital status. A further harmonised variable related to socio-economic status (SES) was defined considering a combination of original variables on educational level and work done for most of the time.
Health status variables	Hypertension, diabetes, hyperlipidaemia, cardiovascular diseases (angina, ischemic heart disease, arrhythmia, peripheral artery disease, stroke), bowel or stomach diseases, liver or gallbladder diseases, chronic bronchitis or emphysema or asthma, cancer, bone or joint diseases, heart failure, depressive symptoms, number of medications.
Neurocognitive outcomes	Cognitive performance (Mini-Mental State Examination) at the baseline, at the first and the second follow-up. Diagnosis of dementia at the baseline, at the first and the second follow-up.
Lifestyle	Smoking habit, alcohol consumption, physical activity, mobility limitations.

Supplementary Table 2. Food groups intake distribution (g/day), by study

	Overall cohort (n=9,016)		BEST-FU (n=1,353)		Pro.V.A. (n=2,981)		ILSA (n=4,428)		NutBrain (n=254)		
	**mean±SD**	**median (Q1, Q3)**	**mean±SD**	**median (Q1, Q3)**	**mean±SD**	**median (Q1, Q3)**	**mean±SD**	**median (Q1, Q3)**	**mean±SD**	**median (Q1, Q3)**	**P-value**
Fruits	386.1±403.5	300 (214.3, 364.3)	975.2±691.3	811 (500, 1249.1)	318.7±226.6	300 (214.3, 364.3)	248.4±103.4	300 (150, 300)	333.7±329	297 (244.5, 342)	<0.0001
Vegetables	200.1±160	171.4 (85.7, 279.3)	351.9±228.8	301.1 (217.9, 435.6)	150.4±94	128.6 (90, 192.9)	179.4±136.1	200 (57.1, 257.1)	308±156.8	286.1 (208.1, 405.2)	<0.0001
Potatoes	41.6±37.9	28.6 (24.3, 57.1)	37.4±37	28 (14, 50)	37.1±26.1	28.6 (24.3, 48.6)	45.6±43.9	28.6 (28.6, 57.1)	49.6±43.1	28 (14.5, 86)	<0.0001
Red meat	36.6±75.8	21.4 (12.9, 45)	98.2±85.1	77.4 (44.1, 126.1)	39.9±105.5	30 (15, 45)	15.2±14.2	14.3 (7.1, 21.4)	26.4±22	18.2 (9.4, 40.9)	<0.0001
White meat	44.1±38.4	38.6 (14.3, 57.9)	47.1±42.1	38.5 (21, 63)	65.2±43	57.9 (38.6, 85.7)	29.4±25.2	28.6 (14.3, 42.9)	28.4±18.7	43 (14, 43)	<0.0001
Cured meat	15.8±21.8	7.2 (7.1, 18.9)	41.1±38.2	32.9 (18.9, 52.5)	12.1±12.2	7.1 (7.1, 14.3)	10.1±12.4	7.1 (0, 14.3)	18.7±16.9	14 (7.3, 21.5)	<0.0001
Legumes	26.2±29	21.4 (9.3, 37.1)	12.6±28.2	7 (0, 16.8)	24.3±14.3	21.4 (21.4, 21.4)	31.4±34.7	21.4 (0, 42.9)	34.7±32.9	21 (10.5, 64.5)	<0.0001
Fish and sea products	27.9±27.8	21.4 (10.5, 42.9)	31.1±35.2	21.7 (8.4, 42)	28.9±22.2	21.4 (15, 35.7)	25.7±28.3	21.4 (0, 42.9)	38.6±24.7	32 (21, 52.5)	<0.0001
Tuna in oil[1]	6.8±11.5	0 (0, 14)	6.9±9.1	5.6 (0, 8.8)	na	na	6.9±12.4	0 (0, 21.4)	4.6±4.3	3.5 (3.5, 7)	<0.0001
Dairy products	126.7±146	125 (53.6, 125)	214.8±308.8	200 (0, 250)	141.8±71.2	125 (125, 125)	87.1±69.7	125 (0, 125)	138.4±146.1	120 (0, 258)	<0.0001
Yogurt	9.2±34.7	0 (0, 0)	26±64.7	0 (0, 26.3)	0.3±6.3	0 (0, 0)	8.1±27.7	0 (0, 0)	42.4±51.4	17.5 (0, 53.8)	<0.0001
Cheese	11.9±28.5	0 (0, 14.3)	44.4±47.8	32.8 (18.2, 54.5)	0.6±6.7	0 (0, 0)	7.4±19	0 (0, 14.3)	44.3±32.4	35.3 (22.7, 60.7)	<0.0001
Cereals	140.1±117.4	94.5 (74.2, 167.9)	114.1±82.4	98.7 (61.6, 145.8)	240.6±139.1	214.3 (130, 323.2)	81.9±44.5	80 (45.7, 91.4)	79.5±37.5	80.2 (51.7, 104.5)	<0.0001
Bread and substitutes	120±79.2	100.8 (80, 150)	139.8±130.1	105.3 (68.5, 156.5)	142.3±84.3	125.7 (87.1, 180)	98.7±39.4	100 (80, 130)	112.9±55.6	104 (74.5, 149)	<0.0001
Stuffed pasta[2]	27.6±33.5	18.1 (6.3, 37.7)	29.6±35.4	19.6 (6.3, 41.7)	12±3.1	12.9 (9.6, 14.3)	na	na	17.5±17.4	9.5 (8.8, 26.3)	<0.0001
Egg	14.1±18.2	7.1 (7, 17.1)	22.3±22	21 (7, 31.5)	21.5±22.2	17.1 (8.6, 25.7)	6.2±7.5	7.1 (0, 7.1)	16.1±12.8	16.8 (7, 21.5)	<0.0001
Dried fruit	1.4±7.4	0 (0, 0)	3.5±14.3	0 (0, 0.3)	0.2±2.6	0 (0, 0)	1±4.2	0 (0, 0)	10.3±15.6	4.2 (0, 12.9)	<0.0001
Sweets and snacks	34.3±51.5	22.3 (10.2, 40.4)	53.6±56.3	42 (12.4, 73.5)	32.9±65	21.4 (14.3, 22.9)	27.7±34.9	17.1 (0, 37.1)	59.1±45.9	49.1 (28.4, 74.6)	<0.0001
Fats	5.7±10.9	1.4 (0, 8.6)	8.5±17.8	1.4 (0, 10)	10.2±11.8	7.9 (2.9, 13.6)	1.9±3.8	0 (0, 1.4)	1.6±3	0 (0, 1.4)	<0.0001
Oil	21.9±13.7	20 (12.5, 25)	21.5±17.1	20 (10, 25)	29±16.7	26.4 (19.3, 34.3)	17±6.3	20 (10, 20)	24.2±7.2	25 (25, 25)	<0.0001
Mineral water[1, 2]	1176.3±1290	1000 (500, 1250)	1214.4±1398	1000 (500, 1250)	na	na	na	na	973.2±269.8	900 (900, 1200)	0.3035
Unsweetened beverages	173.3±170.3	125 (50, 271.4)	189±181.2	150 (100, 250)	282±187.1	280 (167.1, 340)	91.7±91.7	57.1 (28.6, 121.4)	210.3±189	157.5 (125, 271.5)	<0.0001
Sugary beverages	33±118.7	0 (0, 0)	69.3±243.4	0 (0, 28)	11.2±41.7	0 (0, 0)	35.5±87.7	0 (0, 28.6)	51.8±109.1	0 (0, 28)	<0.0001
Spirits	192.3±341.5	101 (0, 250)	313.2±594.5	157 (0, 388)	150.9±182.5	100 (0, 200)	187.1±315.7	125 (0, 250)	126.9±161	60 (1, 166)	<0.0001
Sugars	23.9±80.4	10 (4, 29)	29.2±36.4	20 (8.1, 38.2)	45.1±132.5	30 (14.3, 59.3)	7.7±7.7	5.4 (4, 9.7)	15.3±15	12.5 (5, 22.2)	<0.0001

	Overall cohort (n=9,016)		BEST-FU (n=1,353)		Pro.V.A. (n=2,981)		ILSA (n=4,428)		NutBrain (n=254)		P-value
	**mean±SD**	**median (Q1, Q3)**	**mean±SD**	**median (Q1, Q3)**	**mean±SD**	**median (Q1, Q3)**	**mean±SD**	**median (Q1, Q3)**	**mean±SD**	**median (Q1, Q3)**	
Salt and spices	8.9±21.8	5.1 (4, 10)	7.1±6.8	5.4 (3.1, 10)	159.9±89.7	142.9 (72.9, 250)	na	na	4.9±2.8	4 (4, 5)	<0.0001
Dietetic products	1.5±9.3	0 (0, 0)	0±0	0 (0, 0)	1±4.3	0 (0, 0)	2.5±12.8	0 (0, 0)	0.1±0.5	0 (0, 0)	<0.0001

Abbreviations: na (not available); Q1 (Quartile 1); Q3 (Quartile 3)

Supplementary Table 3. Food groups intake distribution (g/day), by sex

	Women (n=4,745, 52.6%)		Men (n=4,271, 47.4%)		
	**mean±SD**	**median (Q1, Q3)**	**mean±SD**	**median (Q1, Q3)**	**P-value**
Fruits	397.6±430.4	300 (214.3, 383.8)	373.4±371.1	300 (214.3, 310)	0.0404
Vegetables	200.7±166.6	171.4 (85.7, 274.2)	199.5±152.4	180 (85.7, 285.7)	0.6649
Potatoes	39.3±36.5	28.6 (21, 57.1)	44.2±39.2	28.6 (24.3, 57.1)	<0.0001
Red meat	31.9±43.3	20 (9.1, 42.9)	41.7±99.9	21.4 (14.3, 49)	<0.0001
White meat	44.1±37.4	38.6 (18.9, 57.9)	44.1±39.4	35 (14.3, 57.9)	0.0587
Cured meat	15±21.2	7.1 (7.1, 17.1)	16.7±22.4	8.6 (7.1, 21.4)	0.0004
Legumes	24.9±29.5	21.4 (9.3, 28.6)	27.6±28.4	21.4 (9.3, 42.9)	<0.0001
Fish and sea products	26.5±26.3	21.4 (8.6, 39.9)	29.5±29.2	21.4 (11.4, 42.9)	<0.0001
Tuna in oil[1]	6.4±11.2	0 (0, 8.8)	7.2±11.7	0 (0, 16.8)	0.0552
Dairy products	133.7±155.5	125 (89.3, 125)	118.9±134.1	125 (17.9, 125)	<0.0001
Yogurt	11.9±40.6	0 (0, 0)	6.2±26.3	0 (0, 0)	<0.0001
Cheese	11.3±26.4	0 (0, 14.3)	12.5±30.7	0 (0, 14.3)	0.2484
Cereals	134.8±113.3	91.4 (67.3, 163.6)	146±121.4	102.9 (80, 171.4)	<0.0001
Bread and substitutes	112.6±70.5	100 (72.1, 140)	128.1±87.1	108.6 (87, 150)	<0.0001
Stuffed pasta[2]	21.8±26.1	14 (4.9, 30)	33.9±38.9	25.1 (8.8, 46.2)	<0.0001
Egg	14.5±18.3	8.6 (7.1, 17.1)	13.7±18.1	7.1 (7, 17.1)	0.0008
Dried fruit	1.2±6.7	0 (0, 0)	1.7±8	0 (0, 0)	<0.0001
Sweets and snacks	34.9±47.3	22.9 (14, 39)	33.7±55.8	16.6 (8.6, 41.4)	<0.0001
Fats	5.4±9.2	1.4 (0, 8.6)	6±12.5	1.4 (0, 10)	0.0453
Oil	21.6±12.6	20 (12.4, 25.7)	22.3±14.8	20 (12.9, 25)	0.9095
Mineral water[1, 2]	1035.4±984.2	1000 (500, 1200)	1327.1±1538.4	1000 (500, 1350)	0.0001
Unsweetened drinks	182.1±162.4	137.1 (50, 280)	163.6±178.2	100 (50, 250)	<0.0001
Sugary drinks	29.5±97	0 (0, 0)	36.8±138.7	0 (0, 0)	0.2144
Spirits	88.8±158.6	3 (0, 125)	307.4±439.5	202 (61, 401)	<0.0001
Sugars	22.8±30.3	10.3 (4, 30)	25.1±112.3	10 (4.6, 28)	0.8102
Salt and spices[2]	9.7±25.3	5 (4, 10)	8.1±17.1	5.4 (4, 9)	0.6307
Dietetic products	1.7±10.1	0 (0, 0)	1.3±8.3	0 (0, 0)	<0.0001

1: not available for the Pro.V.A. study; 2: not available for the ILSA study

Abbreviations: Q1 (Quartile 1); Q3 (Quartile 3)

Supplementary Table 4. Baseline characteristics, by sex

	Women (n=4,745, 52.6%)	Men (n=4,271, 47.4%)	P-value
Socio-demographic variables			
Education, n (%)			<0.0001
Primary school or less	3730 (79.2)	2834 (66.7)	
Middle school	585 (12.4)	671 (15.8)	
High school	291 (6.2)	477 (11.2)	
University or higher	104 (2.2)	265 (6.2)	
Work done for most of the time, n (%)			<0.0001
Housewife	1323 (29.1)	0 (0.0)	
Blue collar	2084 (45.9)	2674 (63.0)	
White collar	1135 (25.0)	1568 (37.0)	
Marital status, n (%)			<0.0001
Single or never married	397 (8.4)	202 (4.7)	
Married or cohabiting	2067 (43.6)	3408 (79.9)	
Separated or divorced	60 (1.3)	44 (1.0)	
Widowed	2216 (46.8)	610 (14.3)	
Socioeconomic status, n (%)			<0.0001
Low	3497 (73.8)	2626 (61.6)	
Medium	956 (20.2)	966 (22.7)	
High	287 (6.1)	672 (15.8)	
Nutritional status			
BMI, kg/m^2, mean±SD	27.7±4.9	26.6±3.8	<0.0001
Energy intake, kcal, median (Q1, Q3)	2686 (2345, 3052)	2917 (2694, 3440)	<0.0001
Lifestyle and Health status variables			
Smoking status, n (%)			<0.0001
Current smoker	747 (15.8)	843 (19.8)	
Former smoker	445 (9.4)	2353 (55.2)	
Never smoker	3547 (74.9)	1067 (25.0)	
Number of medications ≥5, n (%)	889 (19.7)	584 (14.6)	<0.0001
Mobility limitations, cannot walk, n (%)	206 (6.2)	122 (4.1)	0.0002
Physical activity ≥4 h/week, n (%)	328 (12.7)	486 (24.8)	<0.0001

Q1, Quartile 1; Q3, Quartile 3; SD, Standard Deviation; SES, Socio-Economic Status; na, not available
